# Destruction of the Extensor Mechanism Following an Infected Patellar Fracture Fixation in a Patient With Diabetes: A Report of a Multidisciplinary Case

**DOI:** 10.7759/cureus.89585

**Published:** 2025-08-07

**Authors:** Emmanouil Violakis Georgiladakis, Konstantinos Varvarousis, Andreas Christoforou, Antonis Orphanides, Alexandros Pastroudis

**Affiliations:** 1 Department of Orthopaedics, Asklipieio Voulas General Hospital, Athens, GRC

**Keywords:** diabetic complications, orthopedic implant infection, patellar tendon destruction, patellar tendon necrosis, polymicrobial infection, tension band wiring

## Abstract

Postoperative infections following orthopedic fixation can lead to devastating consequences, particularly in patients with comorbidities such as diabetes mellitus. We present a rare case of a 61-year-old female patient with a patella fracture treated with tension band wiring who developed a severe polymicrobial infection resulting in complete destruction of the patellar tendon. Multiple debridements, removal of implants, and prolonged targeted antibiotic therapy were necessary. Ultimately, a skin graft was required for closure, and a multidisciplinary team recommended against further reconstructive procedures due to high risk. The patient opted to retain limited function without undergoing knee arthrodesis. This case emphasizes the critical importance of early infection control, multidisciplinary coordination, and individualized patient-centered care in complex orthopedic infections.

## Introduction

Patellar fractures represent approximately 1% of all skeletal injuries, most commonly resulting from direct trauma, such as low-energy falls from standing height [1]. The tension band technique remains a widely accepted method for operative fixation due to its favorable biomechanical properties and relatively low complication rates [2]. However, infection remains a significant risk, particularly in patients with comorbidities such as diabetes mellitus, which impairs immune function and delays wound healing [3].

Orthopedic implant-related infections often necessitate hardware removal, extensive surgical debridement, and prolonged antibiotic therapy [4]. In rare and severe cases, deep infections may compromise the extensor mechanism, leading to substantial functional deficits. Reconstruction of the patellar tendon is technically demanding and prone to complications, especially in immunocompromised patients with poor soft tissue envelopes [5].

We present the case of an elderly patient with diabetes who developed deep infection following surgical fixation of a patellar fracture, ultimately leading to destruction of the patellar tendon. The complexity of management necessitated a multidisciplinary team approach, involving orthopedic surgeons, infectious disease specialists, cardiologists, and endocrinologists. This case highlights the ethical and clinical challenges of balancing limb function, quality of life, and surgical decision-making.

## Case presentation

A 61-year-old female patient presented to the emergency department of our tertiary care center on June 14, three days after experiencing a fall from standing height. The patient described the incident as an unassisted mechanical fall occurring at home, without preceding symptoms such as dizziness or syncope. She reported immediate onset of anterior knee pain, swelling, and an inability to bear weight or actively extend the left knee.

**Figure 1:**
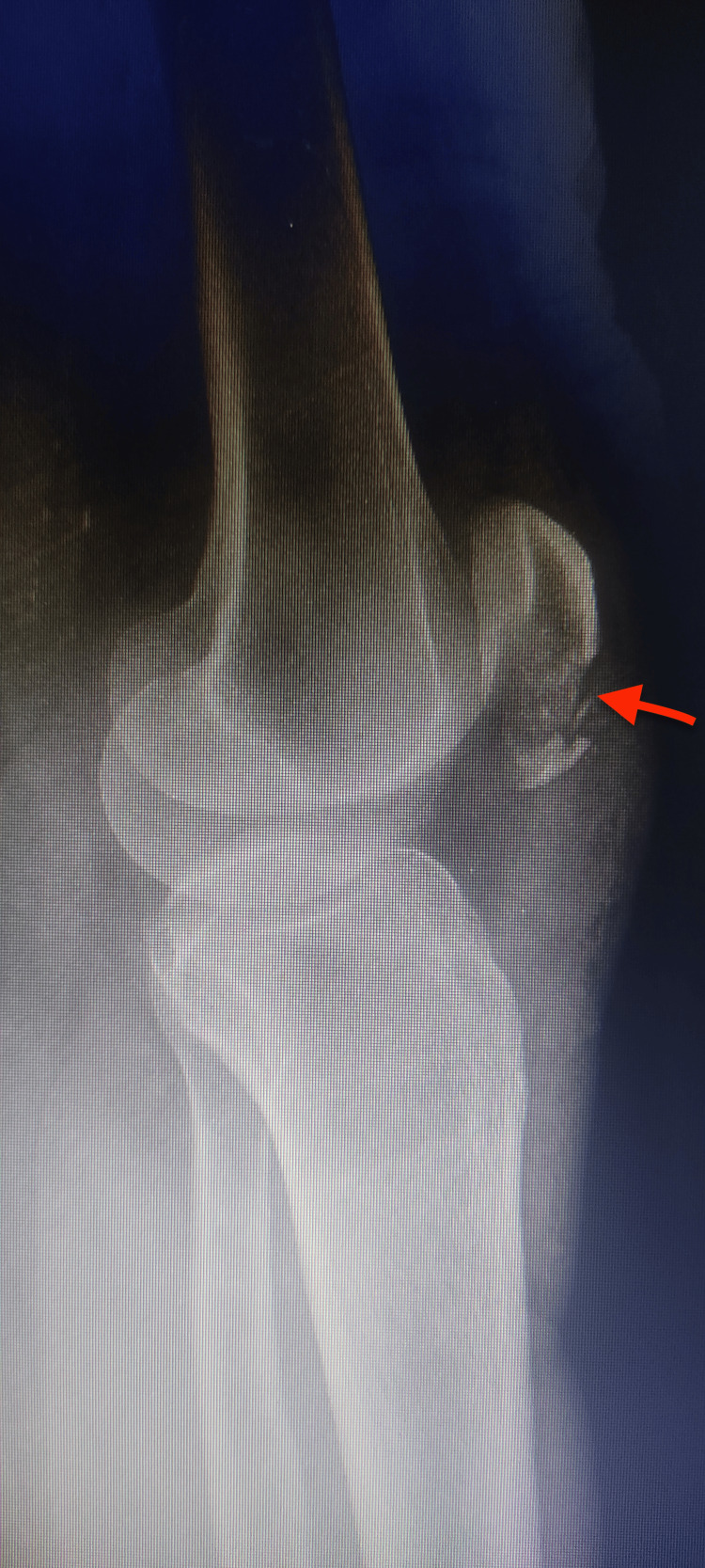
Lateral radiograph of the left knee showing a comminuted inferior pole patellar fracture.

Physical examination showed localized swelling, ecchymosis, and point tenderness over the anterior aspect of the left knee. Palpation revealed a palpable gap over the patella, and active extension of the knee was absent, consistent with extensor mechanism disruption. Neurovascular examination was unremarkable. Standard anteroposterior and lateral X-rays of the left knee confirmed the presence of a displaced transverse fracture of the patella (Figure 1).

A comprehensive review of the patient’s medical history revealed poorly controlled type 2 diabetes mellitus, managed with oral hypoglycemic agents, and long-standing systemic arterial hypertension, which was untreated at the time of admission. The patient also had a significant smoking history, consuming approximately 20 cigarettes per day for over three decades. Her prior surgical history included a cesarean section and a laparoscopic cholecystectomy, both performed over 30 years earlier without complications.

**Figure 2:**
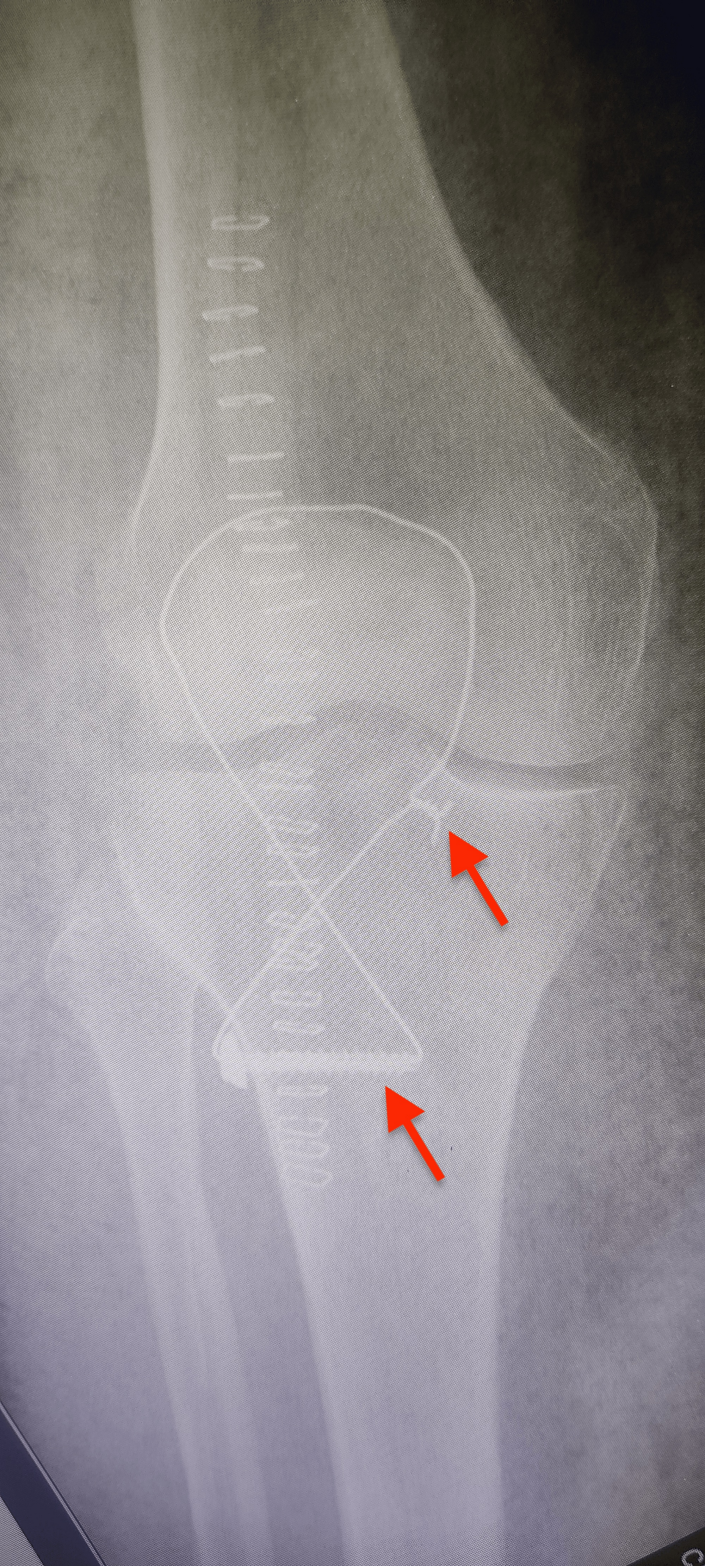
Postoperative anteroposterior X-ray of the left knee after partial patellectomy and fixation with tension band and tibial screw.

**Figure 3:**
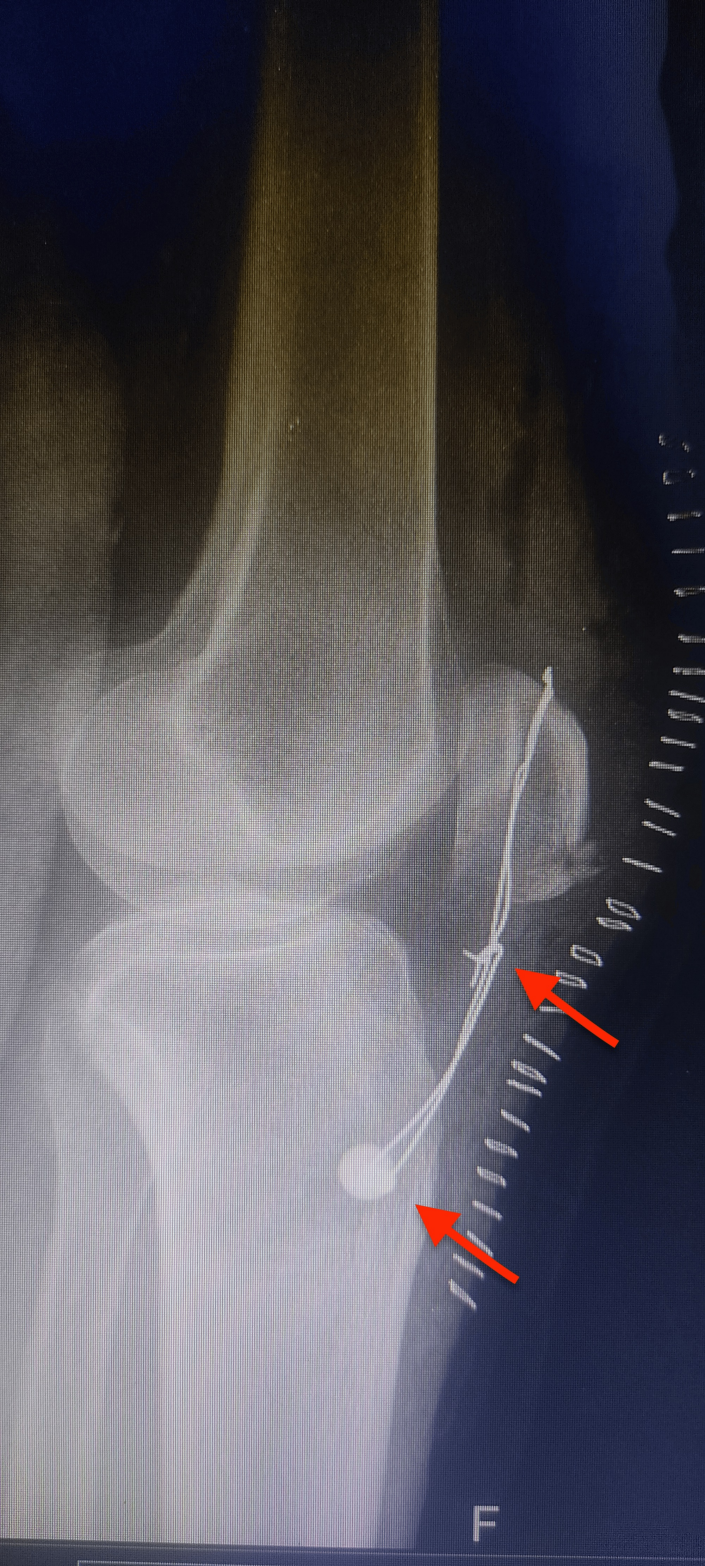
Lateral X-ray of the left knee after partial patellectomy with screw fixation to tibia and tension band construct.

Following multidisciplinary preoperative assessment and optimization of her medical status, the patient was admitted to the orthopedic ward. She subsequently underwent surgery with open reduction and internal fixation (ORIF) of the patellar fracture on the day after presentation, June 15, the fourth day post trauma. Operative exploration revealed a comminuted fracture involving the inferior aspect of the patella. Given the degree of comminution and the inability to achieve stable fixation with tension band wiring alone, the decision was made to perform a partial patellectomy, excising the severely fragmented inferior pole. The remaining proximal patellar fragment was then meticulously prepared, and the patellar tendon was reattached directly to the residual patellar surface using non-absorbable transosseous sutures in combination with a modified tension band technique, as described in the AO principles of fracture management. To augment the construct and improve load-sharing, a cancellous screw was inserted into the anterior tibial metaphysis, anchoring the repair and facilitating biomechanical stability of the extensor mechanism during early mobilization (Figures 2, 3). The procedure was performed under regional anesthesia without intraoperative complications. Initial postoperative recovery was uneventful. The patient was mobilized with a hinged knee brace and instructed to remain partial weight-bearing. She was discharged home on the third postoperative day, June 18, with outpatient follow-up scheduled for suture removal and wound inspection.

First complication: development of postoperative surgical site infection

During the routine outpatient review on the eighth postoperative day, June 23, five days post discharge, the patient presented with increasing erythema, local warmth, and serosanguinous discharge from the surgical wound. Mild tenderness and early signs of wound dehiscence were noted, although there were no initial systemic indicators of infection. A wound swab was obtained for microbiological analysis, and empirical oral antibiotic therapy was initiated while awaiting culture results.

The patient returned on the 13th postoperative day, June 28, with worsening local symptoms and new systemic complaints, including malaise and low-grade fever. Physical examination at that time demonstrated increased wound drainage, surrounding cellulitis, and mild induration. Laboratory evaluation demonstrated leukocytosis (WBC: 16,400/μl) and a markedly increased C-reactive protein (CRP) level (63 mg/L), consistent with systemic inflammation.

Initial culture results from the outpatient wound swab identified *Enterobacter cloacae* and *Staphylococcus epidermidis*, both recognized pathogens in postoperative orthopedic infections. *S. epidermidis*, the most commonly isolated coagulase-negative staphylococcus, is particularly associated with periprosthetic joint infections due to its ability to adhere to implant surfaces and form biofilms. Notably, an unexpected rise in *E. cloacae* infections has been reported in recent years following ankle fracture fixation, with its incidence reaching up to 32.3% when polymicrobial cases are included [6-8]. Given the progression of the infection and the presence of implant material, an urgent surgical intervention was planned. On the 14th day after the first operation, June 29, the patient underwent a comprehensive surgical procedure, including formal wound debridement, complete removal of all implanted hardware, open arthrotomy, and extensive synovectomy. Tissue specimens and cultures were obtained intraoperatively and submitted for microbiological analysis. The cultures reconfirmed the presence of *E. cloacae*, and additionally isolated *Staphylococcus capitis*.

In close collaboration with the Infectious Diseases Department, intravenous antimicrobial therapy was initiated postoperatively. Based on antimicrobial susceptibility testing, the patient was started on a combination regimen consisting of daptomycin 500 mg once daily and meropenem 1 g every eight hours. This regimen was selected to ensure broad-spectrum coverage against the identified gram-negative and gram-positive organisms, including those potentially involved in biofilm formation on orthopedic implants [8].

Second complication: progressive soft tissue necrosis and extensor mechanism failure

Despite the initial improvement in systemic inflammatory markers and reduction in local infection signs, serial wound assessments over the ensuing days revealed ongoing tissue necrosis, particularly over the anterior knee. The skin margins were friable, and there was progressive undermining at the surgical site. Given the concern for persistent deep infection and compromised soft tissue viability, a second surgical debridement was scheduled and performed on July 8, postoperative day nine following the second surgery.

**Figure 4:**
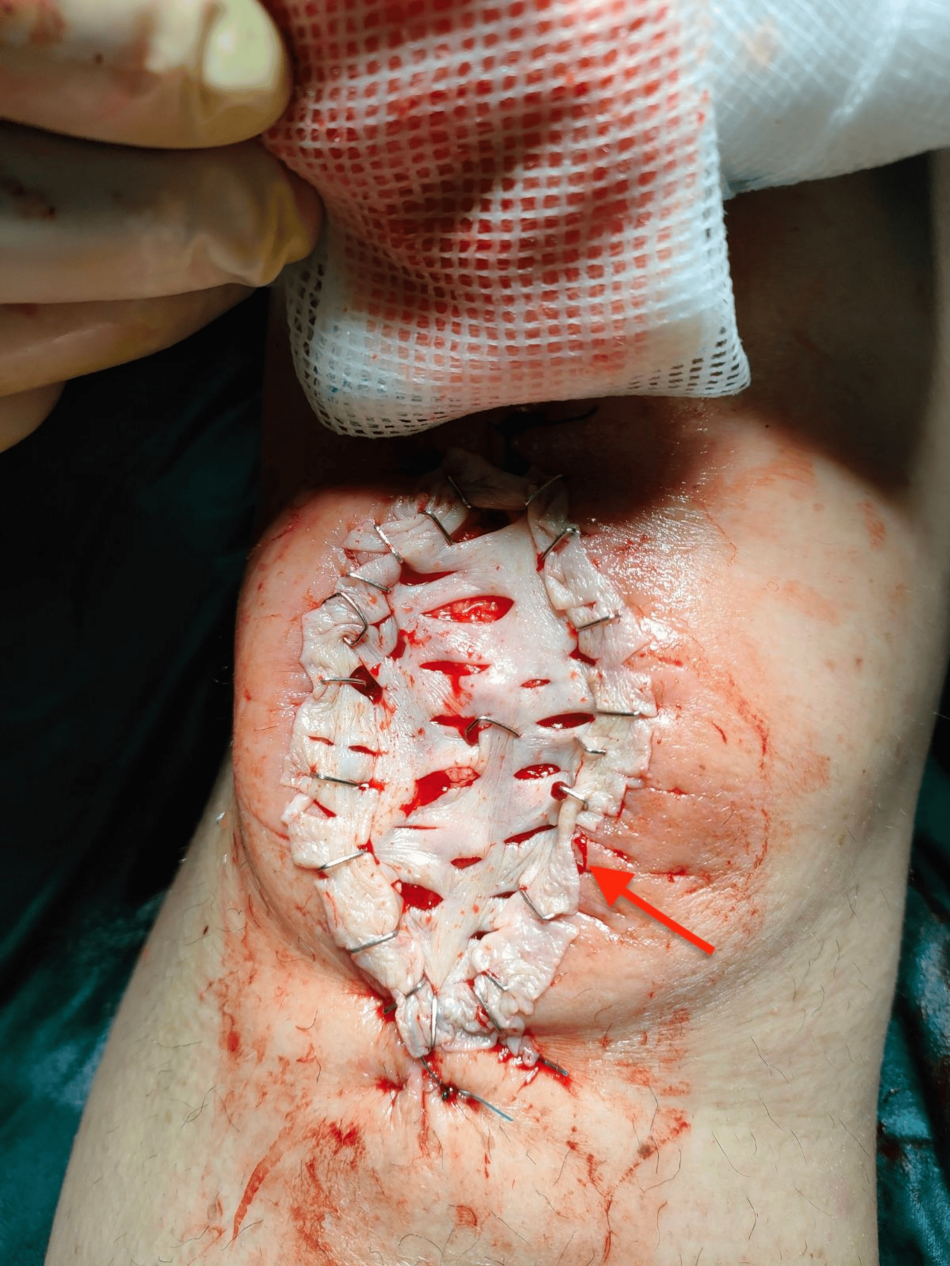
Split-thickness skin graft in place over the anterior knee defect post-debridement.

During the procedure, extensive necrosis of the soft tissue envelope was confirmed, including complete destruction of the patellar tendon. The extensor mechanism was deemed non-reconstructable in the current setting due to the severity of the infection and tissue loss. All devitalized tissue was excised, and a split-thickness skin graft, harvested from the ipsilateral thigh, was applied to the anterior knee defect during the same procedure to facilitate wound coverage (Figure 4). 

During the inpatient period, the patient also received daily physiotherapy sessions aimed at maintaining joint mobility and preventing further muscle atrophy. Passive range-of-motion exercises were initiated early using a continuous passive motion (CPM) device to promote gentle mobilization of the knee joint without stressing the healing soft tissues. Additionally, a tailored program of bedside exercises was implemented under the supervision of a physiotherapist to improve quadriceps activation and preserve lower limb strength. These interventions were critical in maintaining functional potential and preparing the patient for eventual ambulation following wound healing.

**Figure 5:**
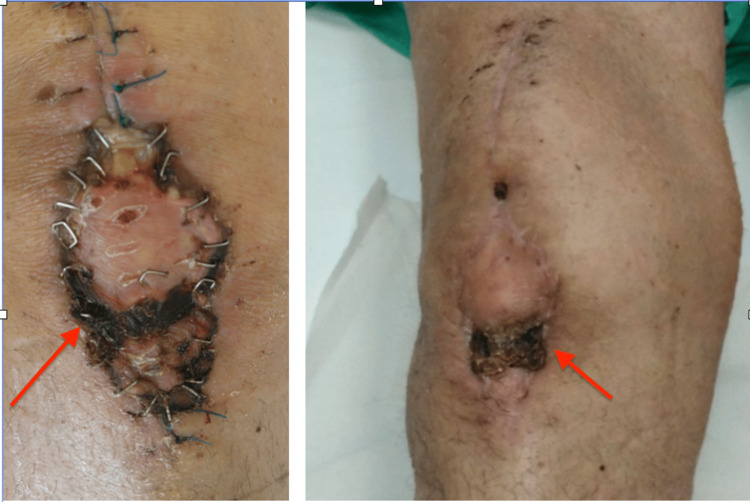
Postoperative follow-up demonstrating excellent take of the split-thickness skin graft with complete epithelialization of the anterior knee defect.

The patient remained hospitalized for continued intravenous antibiotic therapy and close monitoring of glycemic status and cardiovascular function. Endocrinology was consulted and initiated a basal-bolus insulin regimen, which resulted in substantial improvement in glycemic control. Simultaneously, Cardiology managed her previously untreated hypertension with appropriate pharmacologic therapy, and a baseline echocardiographic evaluation was performed. The skin graft demonstrated excellent take, and serial laboratory values showed a gradual normalization of inflammatory parameters (Figure 5).

By mid-August, 30 days following the second debridement, the patient’s wound had healed well, and no further signs of active infection were present. She was discharged on August 16 with instructions for outpatient physical therapy, wound care, and continuation of targeted oral antibiotic therapy as advised by the infectious diseases specialists.

Table 1 summarizes the laboratory markers of inflammation, WBC, and CRP throughout the patient’s hospitalization and recovery period, illustrating the course of infection and response to treatment.

**Table 1:** Laboratory values and clinical course CRP: C-reactive protein

Date	WBC (K/μl)	CRP (mg/L)	Clinical Event / Notes
June 14	15.20	–	Initial presentation – Admission
June 16	13.00	–	Postoperative day 1
June 28	14.50	63	First complication – Early signs of infection
June 30	16.40	–	Hospitalization – clinical worsening
July 2	9.69	66.3	Post first surgical debridement
July 4	10.40	29.4	-
July 9	14.90	-	Second surgical debridement / Monitoring
July 12	9.37	18.3	-
July 15	9.82	13.4	Gradual improvement
July 20	10.40	5.2	Continued improvement, CRP decreasing
July 26	9.80	3.2	Infection resolving
August 2	7.70	3.7	Complete infection healing
August 11	7.12	3.7	Final follow-up – stable condition

Functional status and multidisciplinary decision-making 

At her six-week follow-up appointment, the patient reported moderate improvement in knee comfort and general mobility. Physical examination revealed preserved passive flexion of the left knee up to 60 degrees. Active extension was partially possible up to 30 degrees, despite the functional absence of the extensor mechanism. She was able to ambulate short distances with the assistance of a walker and expressed satisfaction with her overall recovery.

Given the significant functional limitation and loss of the patellar tendon, a multidisciplinary case conference was convened. The team included orthopedic surgeons, infectious disease consultants, endocrinologists, and physical medicine and rehabilitation specialists. Multiple reconstructive options were considered, including extensor mechanism reconstruction using autologous hamstring grafts, Achilles tendon allografts, or synthetic mesh reinforcement [9]. However, these options were ultimately deemed high-risk and inadvisable due to several contributing factors: compromised soft tissue integrity, previous polymicrobial infection, poor host immune status, and elevated risk of recurrence or graft failure.

**Figure 6:**
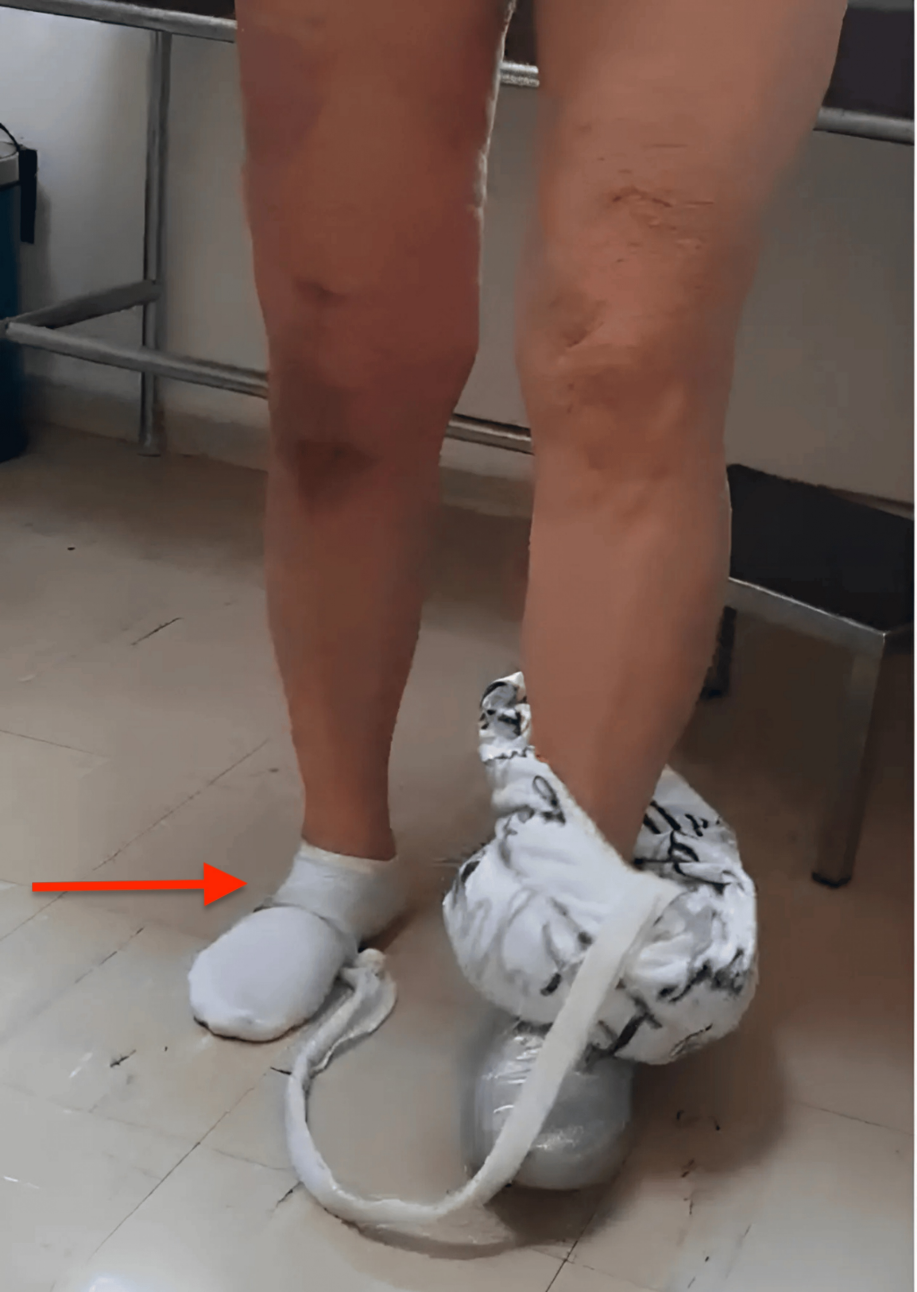
Final follow-up showing single-leg stance

Knee arthrodesis was proposed as a salvage option to provide a stable, pain-free limb [10]. However, after a thorough discussion regarding the functional implications of fusion, the patient expressed a strong preference to avoid any additional surgical interventions. She prioritized preserving her residual knee mobility, even if limited, over pursuing definitive fusion. Her decision was respected in alignment with the principles of patient-centered care and shared decision-making [11].   At the latest follow-up, the patient demonstrated partial restoration of active knee extension with a residual extension lag of approximately 30 degrees and single-leg stand (Figure 6). Despite this limitation, she expressed high satisfaction with the overall outcome, particularly given the severity of the initial complication. Importantly, the patient successfully returned to her professional duties, which primarily involved sedentary office-based work, without significant functional restrictions in daily life.

## Discussion

This case underscores the severe consequences of postoperative infections in orthopedic trauma, particularly among patients with comorbidities such as diabetes mellitus. Surgical site infections in diabetic individuals have been shown to occur at higher rates due to impaired leukocyte function, microvascular disease, and delayed wound healing [12]. Once hardware becomes colonized by bacteria, particularly biofilm-forming organisms such as *S. epidermidis*, conservative treatment alone is often ineffective, and surgical debridement with implant removal becomes necessary [13].

Our patient’s infection involved both gram-negative and gram-positive pathogens. Polymicrobial infections further complicate the clinical course due to the synergistic virulence of organisms and potential multidrug resistance. In this context, timely collaboration with infectious disease specialists was critical to optimizing antimicrobial coverage and treatment duration.

The development of full-thickness patellar tendon necrosis, although rare, is a catastrophic outcome. The patellar tendon plays an indispensable role in knee extension, and its loss results in significant disability. While reconstructive options exist, including the use of Achilles tendon allografts, autologous semitendinosus transfers, or synthetic grafts, each carries a high risk of failure, especially in previously infected or poorly vascularized tissue beds [14,15].

Studies have shown that long-term outcomes of extensor mechanism reconstructions are mixed, with consistently high rates of complications and reoperations reported regardless of the reconstruction technique used [16]. In this case, the decision to defer reconstruction was justified by the high probability of graft failure and further morbidity. Similarly, knee arthrodesis, although reliable for pain relief and stability, drastically limits mobility and significantly impacts the quality of life in ambulatory patients [17].

An essential aspect of this case was the ethical and practical decision-making process. Multidisciplinary input enabled the formulation of a holistic management plan that considered the patient’s comorbidities, functional expectations, and psychosocial context. The patient's informed decision to decline further surgery, despite limited function, highlights the importance of shared decision-making in contemporary orthopedic practice [18].

## Conclusions

This case highlights the complex and multifactorial nature of managing infected orthopedic implants in high-risk patients. Diabetes mellitus significantly increases susceptibility to surgical site infections and impairs the body’s ability to contain infection and heal. In such patients, early recognition, prompt debridement, and multidisciplinary coordination are essential for optimizing outcomes. The destruction of the extensor mechanism represents a rare but devastating complication that leaves few viable surgical options, especially when complicated by soft tissue loss and systemic illness. While reconstructive procedures may offer potential for functional restoration, they are not universally appropriate or effective.

Ultimately, management must be tailored to individual patient needs, risks, and preferences. Shared decision-making and respect for patient autonomy are paramount, particularly when aggressive surgical options offer limited incremental benefit. This case reinforces the principle that, in complex orthopedic infections, successful outcomes extend beyond anatomical repair· they depend on holistic, patient-centered care. It also highlights the fact that, in selected patients, preserving partial joint function may provide a favorable balance between surgical risk and postoperative quality of life.

## References

[1] Melvin JS, Mehta S (2011). Patellar fractures in adults. J Am Acad Orthop Surg.

[2] Nerlich M, Weigel B (2001). Patellar fractures. AO Principles of Fracture Management.

[3] Arshad S, Rasul A, Batool M, Zukhruf Z, Asad MT (2025). Diabetes and risk of surgical site infection: a narrative review. J Health Rehab Res.

[4] Zimmerli W, Trampuz A, Ochsner PE (2004). Prosthetic-joint infections. N Engl J Med.

[5] Brown NM, Murray T, Sporer SM, Wetters N, Berger RA, Della Valle CJ (2015). Extensor mechanism allograft reconstruction for extensor mechanism failure following total knee arthroplasty. J Bone Joint Surg Am.

[6] García Cardona C, Bernaus Johnson MC, Martínez Ros J (2023). Enterobacter cloacae infection after surgical treatment of ankle fractures, a multicenter observational study. Foot Ankle Int.

[7] Brown NM, Murray T, Sporer SM (2025). Bacterial pathogens in orthopedic implant infection and their resistance to antimicrobial therapy: a retrospective analysis. J Orthop Rep.

[8] Tande AJ, Patel R (2014). Prosthetic joint infection. Clin Microbiol Rev.

[9] Kim WT, Kao D, O'Connell R, Patel NK, Vap A (2022). Clinical outcomes are similar between graft types used in chronic patellar tendon reconstruction: a systematic review. Arthrosc Sports Med Rehabil.

[10] Wiedel JD (2002). Salvage of infected total knee fusion: the last option. Clin Orthop Relat Res.

[11] Barry MJ, Edgman-Levitan S (2012). Shared decision making--pinnacle of patient-centered care. N Engl J Med.

[12] Holt RI, Cockram CS, Ma RC, Luk AO (2024). Diabetes and infection: review of the epidemiology, mechanisms and principles of treatment. Diabetologia.

[13] Staats A, Li D, Sullivan AC, Stoodley P (2021). Biofilm formation in periprosthetic joint infections. Ann Jt.

[14] Gencarelli P Jr, Yawman JP, Tang A (2023). Extensor mechanism reconstruction after total knee arthroplasty with allograft versus synthetic mesh: a multicenter retrospective cohort. J Am Acad Orthop Surg.

[15] Diaz-Ledezma C, Orozco FR, Delasotta LA, Lichstein PM, Post ZD, Ong AC (2014). Extensor mechanism reconstruction with achilles tendon allograft in TKA: results of an abbreviate rehabilitation protocol. J Arthroplasty.

[16] Rodríguez-Merchán EC, Encinas-Ullán CA, Ruiz-Pérez JS, Gómez-Cardero P (2024). Chronic extensor mechanism failure after primary or revision total knee arthroplasty: reconstructive and augmentation options. Advances in Revision Total Knee Arthroplasty.

[17] Gramlich Y, Steinkohl D, Kremer M, Kemmerer M, Hoffmann R, Klug A (2021). Modular knee arthrodesis secures limb, mobility, improves quality of life, and leads to high infection control in periprosthetic knee infection, when revision knee arthroplasty is not an option. Arch Orthop Trauma Surg.

[18] Elwyn G, Frosch D, Thomson R (2012). Shared decision making: a model for clinical practice. J Gen Intern Med.

